# Comparing Nadir and Oblique Thermal Imagery in UAV-Based 3D Crop Water Stress Index Applications for Precision Viticulture with LiDAR Validation

**DOI:** 10.3390/s23208625

**Published:** 2023-10-21

**Authors:** Thomas Buunk, Sergio Vélez, Mar Ariza-Sentís, João Valente

**Affiliations:** 1 Laboratory of Geo-Information Sciences and Remote Sensing, Wageningen University & Research, 6708 PB Wageningen, The Netherlands; johnthomasbuunk@outlook.com; 2 Information Technology Group, Wageningen University & Research, 6708 PB Wageningen, The Netherlands; mar.arizasentis@wur.nl (M.A.-S.); joao.valente@wur.nl (J.V.)

**Keywords:** precision agriculture, drone, *Vitis vinifera*, multispectral, vineyard, LWIR, orthomosaic, photogrammetry, 3D point cloud, flight configuration

## Abstract

Unmanned Aerial Vehicle (UAV) thermal imagery is rapidly becoming an essential tool in precision agriculture. Its ability to enable widespread crop status assessment is increasingly critical, given escalating water demands and limited resources, which drive the need for optimizing water use and crop yield through well-planned irrigation and vegetation management. Despite advancements in crop assessment methodologies, including the use of vegetation indices, 2D mapping, and 3D point cloud technologies, some aspects remain less understood. For instance, mission plans often capture nadir and oblique images simultaneously, which can be time- and resource-intensive, without a clear understanding of each image type’s impact. This issue is particularly critical for crops with specific growth patterns, such as woody crops, which grow vertically. This research aims to investigate the role of nadir and oblique images in the generation of CWSI (Crop Water Stress Index) maps and CWSI point clouds, that is 2D and 3D products, in woody crops for precision agriculture. To this end, products were generated using Agisoft Metashape, ArcGIS Pro, and CloudCompare to explore the effects of various flight configurations on the final outcome, seeking to identify the most efficient workflow for each remote sensing product. A linear regression analysis reveals that, for generating 2D products (orthomosaics), combining flight angles is redundant, while 3D products (point clouds) are generated equally from nadir and oblique images. Volume calculations show that combining nadir and oblique flights yields the most accurate results for CWSI point clouds compared to LiDAR in terms of geometric representation (R^2^ = 0.72), followed by the nadir flight (R^2^ = 0.68), and, finally, the oblique flight (R^2^ = 0.54). Thus, point clouds offer a fuller perspective of the canopy. To our knowledge, this is the first time that CWSI point clouds have been used for precision viticulture, and this knowledge can aid farm managers, technicians, or UAV pilots in optimizing the capture of UAV image datasets in line with their specific goals.

## 1. Introduction

Precision agriculture (PA) is an innovative and effective approach to improve crop performance, increase economic benefits, and mitigate the environmental impact by enhancing crop-managing practices and limiting the use of pollutants [1]. PA achieves these goals by using a range of advanced technologies and cost-effective solutions that manage the temporal and spatial variability related to agriculture [2]. Precision agriculture combined with remote sensing techniques has been increasingly implemented in viticulture over the last two decades, resulting in a practice called precision viticulture (PV) [3]. Accurate crop monitoring procedures are essential for the successful implementation of PV processes. In this regard, remote sensing has emerged as a valuable approach, capable of providing vast amounts of data that provide valuable insights into various aspects of crop health [4] and management [5]. Remote sensing techniques enable the assessment of spatial patterns in crop biomass [6] and yield through the utilization of diverse vegetation indices [7] in combination with physiological traits. Common applications are Leaf Area Index (LAI) estimation, nutrient deficiency detection, and health status for precise pesticide application [8]

The introduction of Unmanned Aerial Vehicles (UAVs) has significantly boosted PV, complementing the development of satellite and aircraft remote sensing devices [9]. While satellite and aircraft remote sensing devices have improved the spatial and temporal resolution in studying plant features, aircrafts are expensive, and the spatial resolution of satellites is insufficient for PV [2]. In contrast, UAVs offer a cost-effective solution through their capability to carry sensors that provide high spatial and temporal resolution [10], making them ideal for practices within PV. Additionally, PV benefits from the utilization of three-dimensional (3D) point clouds in crop monitoring, beyond the traditional use of two-dimensional (2D) mosaicked imagery from multispectral images [1]. A point cloud is a collection of data-points in a three-dimensional coordinate system. These points are generated by measuring the external surfaces of objects, resulting in a dense set of spatial data-points that correspond to a geodetic reference frame, representing the reflection of light from the visible surfaces of objects, and capturing the detailed structure and characteristics of them [1]. These point clouds can be created directly from laser scanners, typically through a Light Detection and Ranging System (LiDAR) which uses pulsed laser light to measure distances [11]. Alternatively, they can be created through multispectral and thermal imagery using software that relies on the Structure from Motion (SfM) technique [12]. SfM is a photogrammetry technique that creates a 3D structure from multiple 2D images through feature extraction and matching [13]. Moreover, the application of thermal imaging in the agricultural sector has increased in recent years due to advancements in sensors and reduced costs [14]. Infrared thermal imaging is a technique that captures temperature variations without making direct contact or causing any damage to the object. Utilizing the Long-Wave Infrared (LWIR) spectrum, thermal cameras offer enhanced sensitivity to thermal emissions. This capability allows for more accurate temperature readings, especially in agricultural settings. By detecting the infrared radiation emitted from materials, LWIR sensors are able to translate these emissions into visual data, such as heat maps, that display temperature disparities. This capability is invaluable in various sectors, notably in agriculture. Here, it aids in monitoring plant health, detecting water stress, and identifying areas of potential disease or pest infestation [15], and it has emerged as a reliable method for assessing crop water stress, as it captures changes in plant temperature resulting from water deficits. When plants experience water deficits, stomatal closure occurs, leading to a decreased transpiration rate and reduced evaporative cooling, ultimately resulting in an elevation in leaf temperature [16].

The Crop Water Stress Index (CWSI) is an index that is derived from canopy temperature and has been applied to assess water deficit in various crops, including grapevines [17]. The CWSI has become a valuable index in PA and PV due to its capability to efficiently assess crop water stress accurately over substantial areas. Due to the growing demand for water across multiple sectors and the negative effects of climate change on water resources, it has become essential to prioritize the efficient utilization of water for crop productivity. The insights provided through a CWSI analysis can be applied to optimize irrigation scheduling, thereby maximizing water efficiency and crop yield [18]. Traditional, remote sensing CWSI analysis relies on 2D orthomosaics [19,20,21], which have been shown to be reliable in prior studies [22,23,24]; however, they only provide information about the top of the canopy. In contrast, 3D point clouds can offer a more comprehensive representation of the canopy [25,26], providing additional insights into the side of the canopy and its influence on the CWSI calculation.

Although temperature is a crucial metric for crop monitoring, its resolution is typically lower than other sensors. Therefore, integrating multiple sensors is often necessary to enhancing accuracy [27]. The fusion of thermal data with 3D data, resulting in thermal point clouds, has become a useful method in multiple disciplines since it enhances environmental monitoring practices [28]. Thermal point clouds have been successfully implemented in environmental endeavors such as the characterization of forest canopies [29] and assessing the radiation dynamics of soil surfaces [30]. However, most studies about thermal point clouds have focused on different fields, such as energy inspections in buildings [31].

Therefore, this study aims to bridge the existing research gap by exploring the utilization of thermal point clouds within the context of PV. By combining the aforementioned benefits of remote sensing, thermal imaging, and point clouds, there is a potential to further improve existing methodologies for crop water stress analysis using CWSI. To this end, (i) traditional orthomosaics are generated to compare different approaches in a crop water stress analysis; (ii) moreover, this study adopts multiple flight configurations, including nadir and oblique flights, as well as their combination, to investigate the potential impact of flight configuration on CWSI results. Previous research has shown that oblique imagery combined with nadir images is better for generating 3D models [32,33,34]. However, there is limited research on the influence of nadir and oblique multispectral and thermal images on orthomosaics and 3D point clouds when studied separately. These findings will help to determine the most effective workflow for CWSI analysis within the context of PV, considering different data models.

## 2. Materials and Methods

### 2.1. Research Area

**Figure 1:**
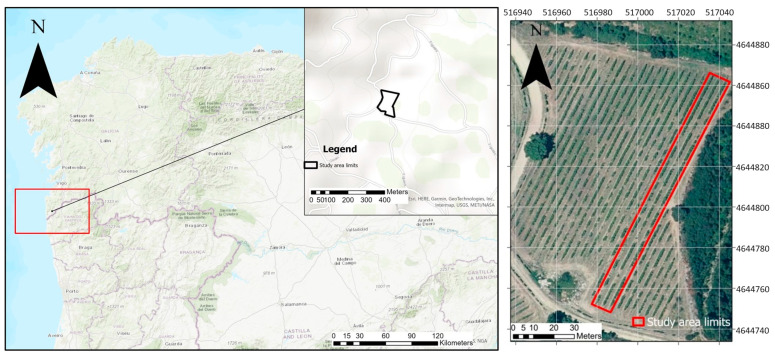
Location of the vineyard. View of the study area with the ROI displayed in red. Coordinates in ETRS89 UTM Zone 29N.

The data were collected from a commercial vineyard (Figure 1) located within “Rias Baixas AOP”, in Tomiño, Pontevedra, Galicia, Spain (X: 516,989.02, Y: 4,644,806.53; ETRS89/UTM zone 29N).

The vineyard, *Vitis vinifera* cv. Loureiro, dates back to 1990 and is part of the ‘Terras Gauda Winery’. The vines were planted with 2.5 m between plants and 3 m between rows with a NE–SW orientation, with cover crops between the vine rows. They were planted using the Vertical Shoot Position (VSP) system, where vine shoots are trained upward with the fruiting zone below. The plants were grafted on a 196.17C rootstock, which is resistant to active limestone. The Appellation of Origin (AOP) rules and practices apply to this vineyard.

### 2.2. LiDAR Data, Multispectral and Thermal Imagery

The LiDAR data were collected using a LiDAR DJI Zenmuse L1 V (DJI Sciences and Technologies Ltd., Shenzhen, China) sensor mounted on a UAV DJI M300 RTK (DJI Sciences and Technologies Ltd., Shenzhen, China), which integrates a flight control system and an FPV camera. It allows obstacle avoidance and six-direction positioning.

The same UAV was used to acquire the multispectral images and thermal data, but was equipped with a MicaSense Altum-PT (AgEagle Sensor Systems Inc., Wichita, KA, USA) to gather the images. The camera captures images in seven different bandwidths (Table 1), geotagging the images using the WGS 84 (World Geodetic System 1984) coordinate reference system (CRS). The camera provides accurate radiometric results with a 1.2 cm pan-sharpened ground resolution when flying at 60 m. The flights were conducted on the 13 July 2022, with two different specifications. The first flight, captured at a nadir angle (0°), started at 12:26 and finished at 12:52, maintaining a flight height of 30 m. The second flight, captured at an oblique angle (30°), started at 16:27 and finished at 16:51, also at a flight height of 30 m. These flight specifications provide crucial details regarding the angles, dates, times, and heights at which the data were acquired.

Both flights were performed with an overlap of 70%. The ground control points (GCPs) were located in the vineyard and georeferenced using the Trimble R^2^ Integrated GNSS system (Trimble Inc., Sunnyvale, CA, USA) to improve the geometric accuracy of the image mosaicking process. In total, 758 nadir and 712 oblique multispectral and thermal images were captured from the whole vineyard (Figure 1).

### 2.3. Proposed Methodology

**Figure 2:**
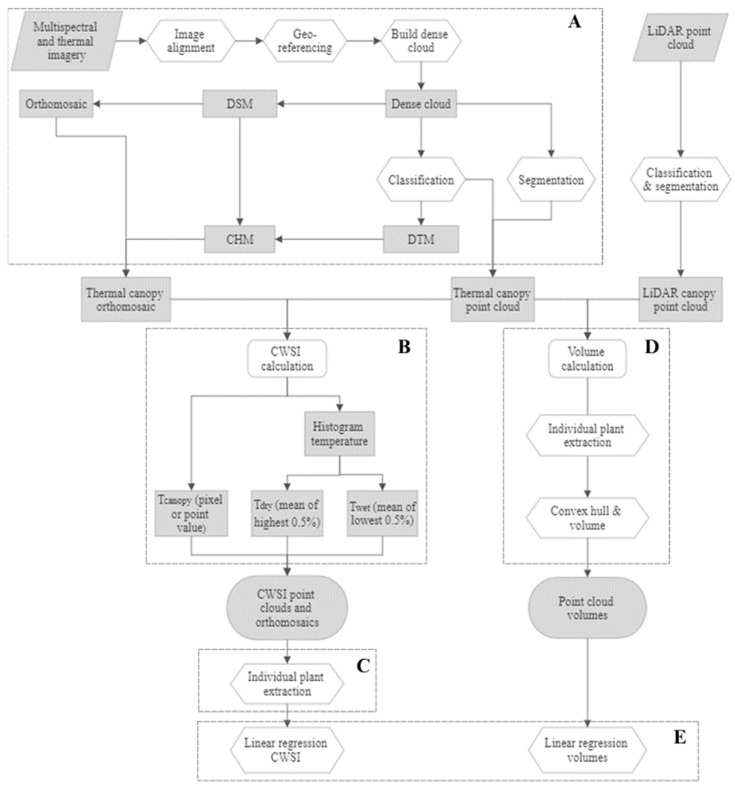
Workflow of the methodology with the different processes represented with a distinct letter. (**A**): image processing; (**B**): CWSI calculation; (**C**): individual plant extraction; (**D**): volume calculation; and (**E**): evaluation methods.

Figure 2 presents the step-by-step workflow of the proposed methodology.

#### 2.3.1. Image Processing (A)

The drone multispectral and thermal images were processed using the Agisoft Metashape Professional v.1.8.5 commercial software (Agisoft LLC., St. Petersburg, Russia). This software utilizes the Structure from Motion (SfM) algorithm, a photogrammetric technique that estimates three-dimensional structures from two-dimensional image sequences. It works by analyzing features in multiple images taken from different viewpoints. By tracking the apparent motion of these features across the image set, the SfM algorithm can deduce the depth and structure of the scene. This technique reconstructs the surface by matching digital images, automatically identifying corresponding points across various orientation operations.

The next step involved the reflectance calibration, using the calibration panel images, and the alignment of the photos (key point limit: 100,000, tie point limit: 10,000), which was achieved through feature detection and matching algorithms that established the relative positions of the images. The result of this step was a sparse point cloud that represented the scene. The sparse point was optimized by employing the gradual selection tool. Outliers of the model were deleted in this step and the photo alignment was optimized after each removal, ensuring a more accurate model. Afterwards, the model was initially converted from WGS84 to the local coordinate system (ETRS89 UTM zone 29N) and then georeferenced with GCPs for a higher accuracy. After georeferencing the sparse point cloud, a dense point cloud of medium quality (as it had been designated by the software) was generated, seeking a balance between quality, time, and computational power, given the high computational requirements for integrating both the nadir and oblique flights. The orthomosaic was then produced by projecting the images onto the Digital Surface Model (DSM).

#### 2.3.2. CWSI Calculation (B)

The CWSI, which relies on canopy temperature, is a widely adopted indicator for assessing plant water stress and mapping spatial variability [35]. Therefore, in this study, the thermal information was isolated in the orthomosaic and in the point clouds, and it was utilized to evaluate the surface temperature of the vineyard’s canopy. To derive accurate temperature values specifically related to the canopy, it was essential to extract pure canopy pixels while eliminating noise sources such as soil and grass. This process involved the creation of a binary mask, utilizing ArcGIS Pro v.3.0.2 (ArcGIS™ software by Esri^®^, Inc., Redlands, CA, USA) based on the Canopy Height Model (CHM). The creation of the CHM involved the generation of a DSM and a Digital Terrain Model (DTM) using the Metashape software. The DSM was generated by incorporating all the points from the point cloud, resulting in a file that represented the height of the various objects within the study area. Simultaneously, the DTM was constructed by initially classifying the ground points within Metashape.

Subsequently, the CHM was derived by subtracting the DTM from the DSM. This process ensured that the CHM specifically captured the vertical extent of the canopy while excluding the ground elevation. Afterwards, a threshold layer was created using a chosen threshold value of 0.5 m to distinguish the cultivated plants from the other objects and plants. In this context, pixels with heights exceeding 0.5 m were assigned a value of one, indicating their association with the canopy, while pixels below this threshold were assigned a value of zero.

Following the generation of the binary mask layer, the thermal layer of the orthomosaic was divided using it. This operation produced a new layer exclusively containing temperature values corresponding to the canopy pixels.

The last step, the CWSI calculation, was performed by applying the following equation:

(1) $$CWSI=\frac{T_{canopy}-T_{wet}}{T_{dry}-T_{wet}}$$

In the CWSI calculation, the measured temperature of the canopy pixels was compared to the lower and upper limits representing a non-stressed leaf (T_wet_) and a leaf in maximum stress (T_dry_), respectively. As discussed previously, various approaches exist for determining the reference temperatures T_wet_ and T_dry_. A modified approach known as the simplified CWSI approach [19,21] was employed. In this method, the temperature histogram was utilized to determine the wet and dry reference values. Specifically, the wet reference was calculated by averaging the lowest 0.5% of the histogram, while the dry reference was obtained by averaging the highest 0.5% of the histogram. This modification enabled a more accurate assessment of water stress by aligning the reference values with the temperature distribution observed in the orthomosaics.

In the thermal point cloud classification, Metashape was employed to discriminate between the canopy and the underlying terrain by automating the ground point classification. This involved dividing the dense cloud into cells, detecting the lowest point per cell, and triangulating these points to create an initial terrain model. Subsequent points within a certain distance and angle threshold were allocated as ground points, with the flexibility to adjust these parameters for an optimized classification.

The classified point cloud, particularly the canopy, was exported as a text file, storing reflectance values in distinct columns, thus streamlining the thermal point cloud analysis. CloudCompare (version 2.12.1), an open-source software, was utilized to import the point cloud. In CloudCompare, the reflectance values were archived as a scalar field within the file to preserve spectral information. The software’s noise filtering capabilities refined the point cloud by eliminating outliers, thus yielding an accurate depiction of the canopy structure.

Manual editing was imperative in the classification phase, where non-canopy elements, such as soil and trunks, were meticulously eliminated. This ensured the point cloud’s fidelity to the actual canopy structure.

Finally, temperature values were converted from Kelvin to Celsius using CloudCompare’s arithmetic tool. These temperature values facilitated the computation of T_wet_ and T_dry_, which served as reference points in calculating the CWSI for each point in the cloud via a specified equation. This culminated in a CWSI point cloud representing water stress levels throughout the study area.

#### 2.3.3. Individual Plant Extraction (C)

**Figure 3:**
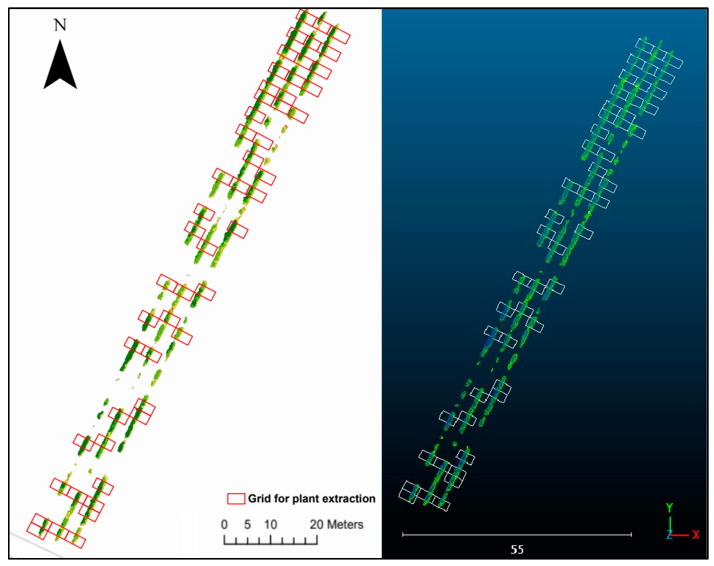
Grids used to isolate the individual plants. The left image illustrates the nadir orthomosaic, while the right image showcases the nadir point cloud. Coordinates are represented in ETRS89 UTM Zone 29N.

Individual plants were isolated using a grid created in ArcGIS Pro, dividing a polygon around the research area into equally sized segments. The selection of segments ensured equal distribution and eliminated small canopy parts to prevent bias, yielding 51 polygons. The same grid was applied to all the orthomosaics and point clouds for consistency. Figure 3 illustrates the grid’s application. While the grid does not perfectly match the individual vines due to size variations, it was created using the in-field plant and row measurements. This approach enables consistent isolation for a comparative analysis between data models.

#### 2.3.4. Volume Calculation (D)

This study utilized both nadir and oblique flight configurations, as well as a combination of the two, to evaluate their impact on CWSI calculation. To assess the geometric precision of the generated point clouds, volume calculations were performed. The plant volumes of each flight were compared to the volumes of an LiDAR dataset which is used for ground-truth comparisons. The volumes were calculated using the convex hull method, which encompasses the plants with the smallest possible convex shape.

#### 2.3.5. Evaluation Methods (E)

Firstly, a comparison was made between the CWSI values of the orthomosaics and those of the point clouds. To assess the similarities and differences between these data models, linear regression was employed. A subsequent analysis involving canopy segmentation explored the contributions of side and bottom canopy portions within the point clouds towards the detected disparities. Additionally, linear regression was performed across the various flight angles to investigate their impact on the CWSI calculation.

Subsequently, the volumes derived from the nadir, oblique, and combined flights were compared to the LiDAR data through linear regression to validate which flight offered the most accurate volume estimation. This evaluated the quality and reliability of the point clouds.

The R software (version 4.2.2, R Foundation for Statistical Computing, R Core Team 2019, Vienna, Austria) was employed to carry out the statistical analyses.

## 3. Results

In the first results’ section, the orthomosaics and point clouds created with ArcGIS Pro, CloudCompare, and Metashape are displayed to highlight the procedure and the CWSI distribution in the study area which could be appreciated visually. The subsequent section contains scatter plots showcasing the outcomes of the linear regression analyses. These plots firstly show regressions between the point clouds and the orthomosaics, aimed at assessing the precision of the point cloud data. Furthermore, regressions are displayed between the varying flight angles to explore how flight orientation affected both the orthomosaics and the point clouds. Lastly, the section encompasses regressions that compare the point cloud volumes extracted from the thermal UAV data against the corresponding volumes derived from the LiDAR data, offering an assessment of the point clouds’ accuracy.

### 3.1. Qualitative Results

#### 3.1.1. Orthomosaics

**Figure 4:**
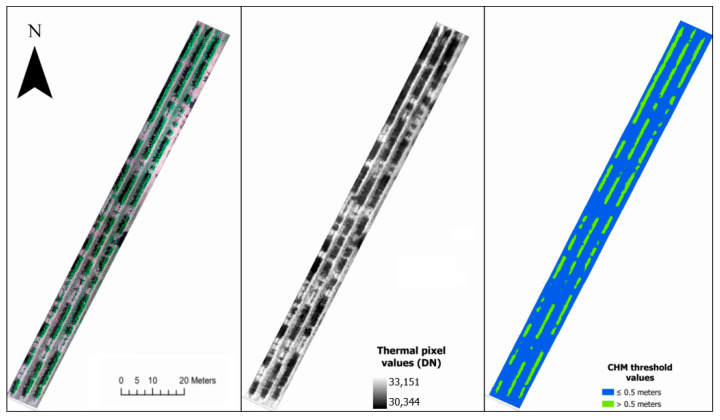
Orthomosaics of the study area from the nadir flight in RGB (**left**), thermal (**center**), and CHM’s threshold (**right**). Coordinates in ETRS89 UTM Zone 29N.

**Figure 5:**
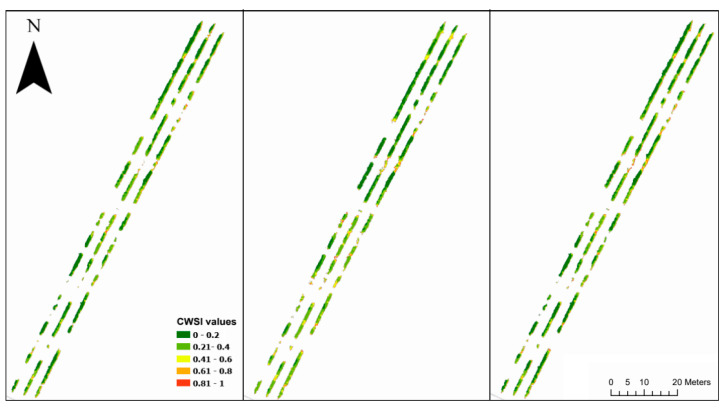
Orthomosaics of the CWSI values of the nadir flight (**left**), the oblique flight (**center**), and the two flights combined (**right**). Coordinates in ETRS89 UTM Zone 29N.

The study area’s detailed orthomosaics from the nadir flight are depicted in Figure 4. It showcases the RGB representation, the thermal imaging, and the Canopy Height Model (CHM)’s threshold layer, all based on the ETRS89 UTM Zone 29N coordinates. The CHM’s threshold layer is instrumental in differentiating between canopy and non-canopy elements, especially in scenarios like this vineyard, where there are missing plants within rows. When the thermal layer is divided using this threshold, it isolates pixels that solely represent the canopy, paving the way for the creation of the CWSI layer. Building on this, Figure 5 provides a comprehensive view of the CWSI orthomosaics. It displays the results from various flight configurations: the nadir flight on the left, the oblique flight in the center, and a combination of both flights on the right. The values of the orthomosaics range from zero to one, with any minor outliers being excluded from the dataset. Across all the flights, a consistent pattern emerges, characterized by lower CWSI values observed in the northern region of the vineyard. Simultaneously, elevated CWSI values can be seen predominantly along the edges of the vine rows, mainly on the eastern side of the vineyard. However, the oblique flight displays heightened stress levels in the southern section of the vineyard, compared to the other two flights.

#### 3.1.2. Point Clouds

**Figure 6:**
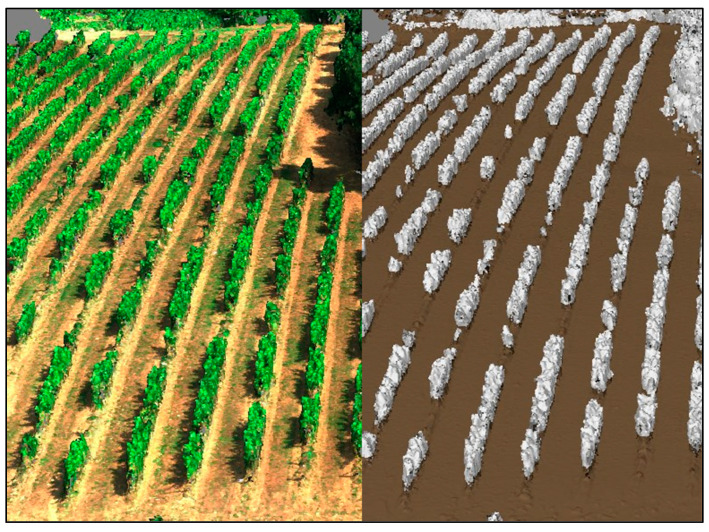
Point clouds of the nadir flight in RGB (**left**) and the ground point classified version (**right**).

Figure 6 presents the RGB point cloud and the classified version from the nadir flight. Both images depict a larger portion of the field beyond the study area, as segmentation had not been performed yet. The image on the right illustrates the outcome of Metashape’s automatic ground point classification function. This function effectively distinguished the ground points (depicted in brown) from the canopy points (depicted in white). Subsequently, the white portion of the point cloud underwent further processing in CloudCompare. After the final segmentation was carried out, the excluded canopy points were used to compute the CWSI point cloud.

**Figure 7:**
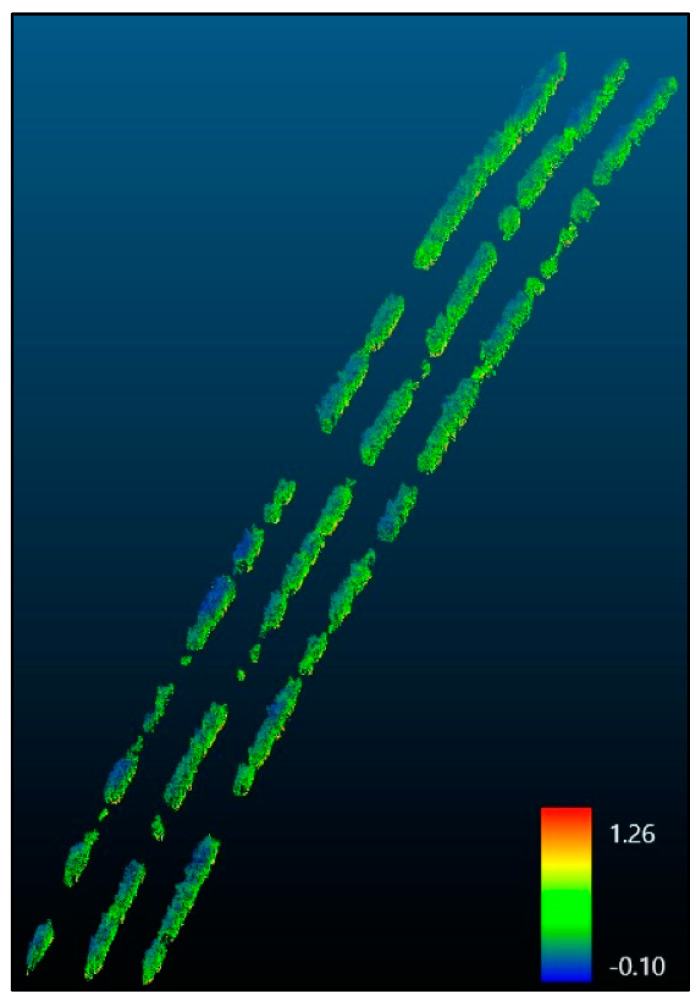
CWSI point cloud of the nadir flight.

Finally, the CWSI point cloud (Figure 7) was produced through further processing. The point cloud displays a pattern with lower CWSI values at the top and higher values towards the bottom of the canopy. Although the legend displays values that exceed the zero to one range, it should be noted that these values constitute outliers, accounting for less than one percent of the entire point cloud, much like the orthomosaics’ outliers.

### 3.2. Statistical Results

#### 3.2.1. General Statistics

Table 2 presents a summary of the data models in terms of the temperature and CWSI values. The oblique flight, with 47,717 pixels in the orthomosaic and 181,372 points in the point cloud, has a lower granularity and coverage compared to the other flight configurations. Notably, the maximum temperature recorded during the oblique flight was approximately forty-four degrees. In contrast, the other two flight configurations recorded maximum temperatures around fifty-four degrees, indicating a difference of about ten degrees.

#### 3.2.2. CWSI Accuracy and Flight Angle Influence

**Figure 8:**
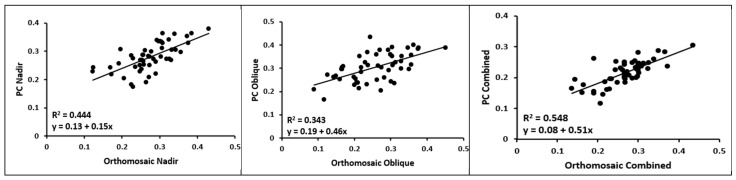
Linear regression between the CWSI orthomosaic and CWSI point cloud values of the nadir flight (left top), the oblique flight (right top), and the combined flights (bottom). PC: point cloud.

**Figure 9:**
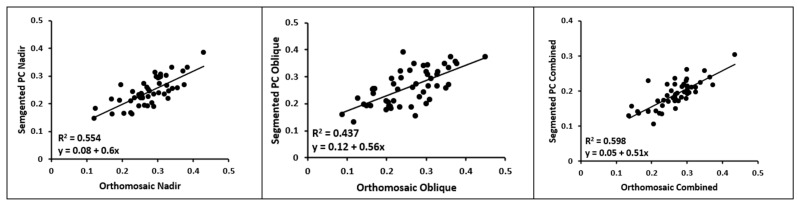
Linear regression between the CWSI orthomosaic and segmented CWSI point cloud values of the nadir flight (left top), the oblique flight (right top), and the combined flights (bottom). PC: point cloud.

Statistical analysis was employed between the mean CWSI values of each plant from each data model and flight. The linear regression results between the orthomosaic and point cloud of each flight configuration are displayed in Figure 8. The low R^2^ values indicate a disparity between the orthomosaics and the point clouds. To further analyze the difference between the data models, the tops of the point clouds were segmented, and linear regression was repeated using only the top portion (Figure 9). These findings indicate that the segmented CWSI point cloud has a stronger correlation with the CWSI orthomosaics compared to the entire point cloud.

**Figure 10:**
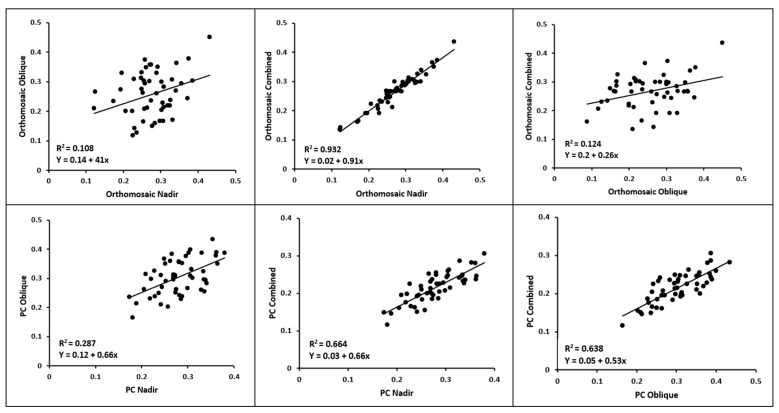
Linear regression between different flights of orthomosaics (**top**) and point clouds (**bottom**). PC: point cloud.

To further explore the variations observed among the different flights and data models, Figure 10 presents the linear regression results comparing flights within the same data model.

The scatter plots depicting the orthomosaics reveal a notable disparity between the oblique and nadir flight configurations, as evidenced by the significantly low R^2^ value. Similarly, the oblique and combined flight configurations also exhibit a considerable difference. Conversely, the nadir and combined flight configurations display a remarkably high R^2^ value, indicating a close resemblance between the datasets.

When analyzing the point clouds, the substantial distinction between the nadir and oblique flights is observed again. The R^2^ values between the nadir and the combined, as well as the oblique and the combined, flights, however, are nearly identical, indicating a more balanced relationship between the datasets.

### 3.3. Point Cloud Accuracy

The subsequent section presents the results obtained from the volume calculations performed on the point clouds derived from the various flights, as well as the LiDAR point cloud. The volume measurements from the LiDAR point cloud are considered ground-truth data due to their high accuracy. While the accuracy of the volume calculations does not directly provide information about the CWSI calculation, it demonstrates how well the point clouds captured the actual geometry of the vineyard. Thus, a more accurate representation of reality leads to more precise results.

The LiDAR point cloud underwent classification and segmentation using the same methodology and parameters as the other point clouds. Through these calculations, the following linear regression results between the point clouds and the LiDAR dataset were obtained.

**Figure 11:**
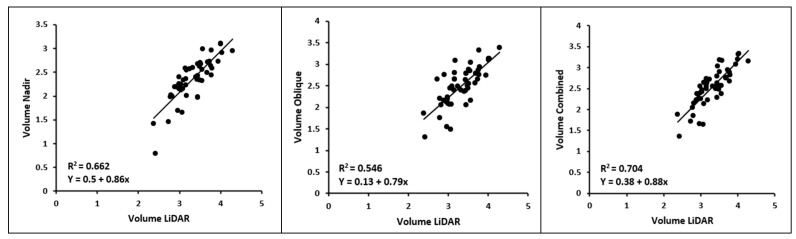
Linear regression between the point cloud volumes of each flight and the LiDAR point cloud volume.

Figure 11 shows that the volume calculations display a relatively strong model fit for the nadir dataset with an R^2^ of 0.66, especially for the combined dataset, with an R^2^ of 0.70. However, the oblique flight by itself shows the lowest correlation with the LiDAR volume, with an R^2^ of 0.55.

Regarding the heights and volumes of the point clouds, they are quite similar, except for the LiDAR point cloud, which displays notably higher values. Despite applying the same classification and segmentation methodology, the average height of the LiDAR point cloud exceeds that of the other datasets by more than 20 cm. Consequently, to improve the model fit, the LiDAR point cloud was further segmented by excluding the bottom 20 cm. This adjustment resulted in the following outcomes.

**Figure 12:**
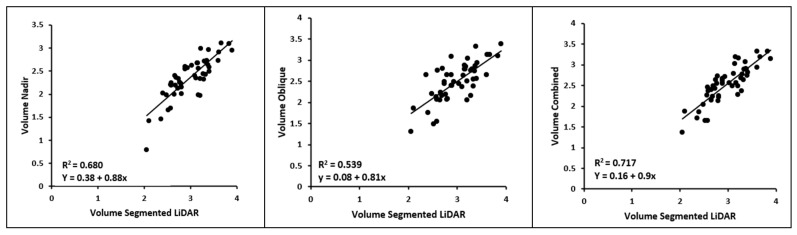
Linear regression between the point cloud volumes of each flight and the volumes of the segmented LiDAR point cloud.

The R^2^ values for the nadir and combined flights indicated an improvement of 0.02 and 0.01, respectively. However, there was a slight decrease of 0.01 in the R^2^ value for the oblique point cloud. Figure 12 illustrates that the combined datasets yield the most accurate volume calculation, making it the most suitable approach for generating a point cloud that closely represents the actual vineyard. The nadir flight demonstrates a similar level of accuracy, while the oblique flight, when considered alone, proves to be the least accurate.

While these volume calculations do not directly reflect the accuracy of the CWSI calculation, they do highlight that the nadir flight is more likely to generate a high-quality point cloud compared to the oblique flight. Moreover, the results show that combining both nadir and oblique flights ensures the best results in terms of point cloud accuracy.

## 4. Discussion

The first inquiry focused on water stress studies employing multispectral and thermal UAV imagery. Prior studies have underscored the utility of these technologies in monitoring water stress in various crops, including vineyards. In this way, Bellvert et al., [36] used CWSI to map water deficits in a vineyard, finding a strong correlation with the leaf water potential when measured with infrared temperature sensors on grapevines and showing that high-resolution airborne thermal imagery was also effective in assessing water stress for precision irrigation. Prueger et al., [37] evaluated CWSI in a vineyard with multiple canopy temperature sensors finding that frequent irrigation maintained the soil water content near field capacity, resulting in decreased CWSI values during peak evaporative demand. Matese et al., [38] compared remote and proximal sensing measurements with plant physiological variables in two vineyards, finding significant correlations between the CWSI and the net photosynthetic rate (Pn) and other physiological parameters, suggesting CWSI as a valuable index for assessing crop water status variability in Mediterranean vineyards. Martínez-Peña et al., [39] found that CWSI correlated significantly with various quality parameters of pistachio trees, surpassing non-thermal indices, suggesting CWSI as a valuable tool for pistachio orchards’ management. This work adds to this literature by zeroing in on thermal point clouds for a nuanced understanding of canopy representation in CWSI analysis.

The second objective concerned generating CWSI orthomosaics and point clouds using thermal UAV data. The results displayed the successful creation and evaluation of these models. The point clouds furnished a more detailed portrayal of the canopy, with lower CWSI values at the top of the canopy (Figure 7). These findings suggest that thermal emissions from the soil contribute to the observed CWSI pattern due to the soil’s higher reflectance rate in the thermal spectrum. The subsequent objectives delved into the contrasts between the data models and the influence of flight angles on the CWSI calculations. The point clouds exhibited a broader range of temperature and CWSI values compared to the orthomosaics, attributable to the additional data they capture. The regression analyses revealed a moderate correlation between the values from the orthomosaics and point clouds. This suggests that point clouds provide supplementary insights by considering the entire canopy structure, which is not obtained through traditional 2D orthomosaics.

A striking observation here was the low R^2^ value when comparing the CWSI values obtained from the nadir and oblique flights during the point cloud analyses. This low R^2^ value suggests a significant variability between these two flight types. Given that both flights were conducted on the same day, one would expect the CWSI values to be more similar. In contrast, the R^2^ values for the combined flights (nadir + combined and oblique + combined) were considerably higher at 0.66 and 0.64, respectively. The similarity of these R^2^ values would suggest that the point cloud derived from the combined flights contained roughly equal contributions from both the nadir and oblique flights. However, a different pattern occurred when considering the orthomosaic datasets. The R^2^ value for the nadir + combined orthomosaic was 0.93, while for the oblique + combined orthomosaic it was only 0.12. This stark difference indicates that the orthomosaic dataset derived from the combined flights predominantly included values obtained from the nadir flight, with a minimal representation from the oblique flight. This implies that, for tasks carried out using 2D analysis, it is practical to employ solely the nadir angle by itself and abstain from using a combination of nadir and oblique flight angles. Combining these two angles in 2D analysis yields results nearly indistinguishable from those obtained using solely the nadir angle. Therefore, opting for a single flight not only saves time but also significantly reduces storage requirements since a multispectral dataset of images from a single flight over the rows of the study could surpass 10GB [40], and over the whole field could exceed 80 GB. Furthermore, the computational demands are significantly higher when using nadir and oblique flights combined. 

For 3D analysis, however, the results illustrate that nadir and oblique flight contribute equally to the output. Consequently, 3D applications, such as the current 3D CWSI analysis, or applications enhanced by considering the entire canopy [41,42], can be improved by using combined angles. This is further supported by the results of the volume analysis. The combined flights exhibited the highest R^2^ value (0.72), surpassing both the nadir flight (0.68) and the oblique flight (0.54). This regression analysis emphasized the superiority of the nadir flight in generating precise point clouds, while also showcasing that the combination of both flights yielded the most optimal results.

This latter finding is in line with previous research that demonstrates that combining nadir and oblique angles leads to a more accurate 3D analysis [43,44]. However, these studies also found that, by itself, the oblique angle outperforms the nadir, a fact which is attributed to its ability to capture a greater number of underlying points. In the present study, however, the oblique flight captured fewer points than the other two workflows. Hence, the effectiveness of the convex hull method for volume calculation could have been diminished in this particular flight, explaining the weaker performance. As [45] showed, this method can lead to overestimation in the presence of gaps within the point cloud.

Finally, Figure 9 demonstrates significant increases for all the flights following the segmentation of the top of the point clouds. This finding suggests that the discrepancy between the orthomosaic and point cloud values is partially attributable to the additional information contained in the point clouds regarding the side and bottom of the canopy. However, despite these improvements, the R^2^ values remained relatively low. This indicates that there are other factors influencing the CWSI calculation when utilizing the point cloud approach.

The results of this study are valuable for real applications since they offer insights into optimizing UAV flights for water stress monitoring in agriculture. For practical farm implementation, before irrigation season, UAVs could be used for a baseline water stress analysis. Then, regular UAV flights should be scheduled during key growth phases for a detailed analysis of the status of the canopy, aiming to adjust irrigation based on the UAV data, targeting areas of high stress. Future studies should incorporate data measured in the field for an improved accuracy assessment. Additionally, employing advanced edge detection algorithms could enhance the segregation of soil and canopy in the point cloud data, leading to a more reliable and accurate analysis. It is also advisable to execute flights simultaneously to minimize discrepancies due to temperature variations, allowing for an accurate appraisal of the flight angle’s specific impact on the CWSI calculations.

## 5. Conclusions

This study investigated the effect of different flight configurations for point cloud generation and the utility of thermal point clouds in precision viticulture for water stress evaluation. Three-dimensional point clouds embedded with CWSI values were generated and juxtaposed with two-dimensional CWSI orthomosaics, yielding insights into the relative merits of these data models. Furthermore, the influence of flight configuration on CWSI computation using point clouds and orthomosaics was also evaluated. In this work, we were able to discern the distinctions between the data models and evaluate the effect of the flight angles on the CWSI computations depending on the generated product (point clouds or orthomosaics). The results showed the following:

- The point clouds unveiled a distribution pattern of the CWSI values throughout the canopy not observable with the orthomosaics. The linear regression analysis revealed that the segmented CWSI point cloud had a stronger correlation with the CWSI orthomosaics compared to the entire point cloud, accentuating the information augmentation provided by the point clouds, especially when considering the entire canopy structure.
- The point clouds manifested a broader range of temperature and CWSI values, attributable to the additional information from the lateral and basal canopy regions.
- Furthermore, nadir images by themselves are adequate for 2D orthomosaic creation; yet, the combination of oblique and nadir imagery is essential for generating accurate 3D point clouds in precision viticulture/precision agriculture for woody crops.
- Finally, the volume calculations revealed that combining the nadir and oblique flights engendered the most accurate results compared to the ground-truth data (LiDAR) in terms of canopy representation, which likely enhanced the accuracy of the CWSI assessments.

To our knowledge, this is the first time that CWSI point clouds have been used for precision viticulture. The results suggest that thermal point clouds possess the potential to improve water stress analysis in precision viticulture, delivering a more exhaustive canopy depiction and helpful insights into the temperature and CWSI distributions, showing that employing nadir and oblique flights combined is optimal for point cloud analysis. These insights can help farm managers and UAV pilots to optimize UAV image datasets for thermal and CWSI point cloud generation, potentially enhancing irrigation management to align with their goals. However, additional research is required to surmount the identified constraints and fine-tune the methodologies for pragmatic incorporation into vineyard management.

## Figures and Tables

**Table 1:** Center wavelengths and bandwidths of the MicaSence Altum-PT.

Name	Center	Bandwidth	Sensor Resolution (Pixels)
Blue	475 nm	32 nm	2064 × 1544 (3.2 MP)
Green	560 nm	27 nm	2064 × 1544 (3.2 MP)
Red	668 nm	16 nm	2064 × 1544 (3.2 MP)
Red edge	717 nm	12 nm	2064 × 1544 (3.2 MP)
Near-infrared	842 nm	57 nm	2064 × 1544 (3.2 MP)
Panchromatic	634.5 nm	463 nm	4112 × 3008 (12 MP)
LWIR (Thermal)	10.5 × 10^3^ nm (10.5 μm)	6 × 10^3^ nm	320 × 256 (0.08 MP)

**Table 2:** General statistics regarding the temperature and CWSI of the three data models (nadir, oblique, and combined).

Flight	Data Model	Min Temp	Max Temp	Mean Temp	St. Dev. Temp	Min CWSI	Max CWSI	Mean CWSI	Twet	Tdry	St. Dev CWSI	Pixels/Points (N)	Pixel Size (cm/Pixel)/Point Distance (cm)
Nadir	Ortho	31.9	54.4	37.9	3.42	−0.04	1.21	0.29	32.5	50.7	0.19	66,945	1.57
	PC	31.7	56.2	38.9	3.69	−0.10	1.26	0.30	33.5	51.5	0.20	215,199	5.58
Oblique	Ortho	31.4	44.8	34.9	1.86	−0.04	1.30	0.31	31.8	41.9	0.19	47,717	1.91
	PC	31.0	43.2	35.2	1.64	−0.09	1.20	0.35	31.9	41.3	0.17	181,372	6.64
Combined	Ortho	31.8	55.5	38.3	3.83	−0.04	1.19	0.30	32.5	51.8	0.20	53,315	1.79
	PC	31.1	55.0	36.4	2.72	−0.06	1.27	0.23	32.2	50.1	0.15	208,687	6.04

## Data Availability

The data that support the findings of this study are available from the corresponding author upon reasonable request.
